# User experience with a parenting chatbot micro intervention

**DOI:** 10.3389/fdgth.2022.989022

**Published:** 2023-01-11

**Authors:** G. A. Entenberg, G. Dosovitsky, S. Aghakhani, K. Mostovoy, N. Carre, Z. Marshall, D. Benfica, S. Mizrahi, A. Testerman, A. Rousseau, G. Lin, E. L. Bunge

**Affiliations:** ^1^ Research Department, Fundación ETCI, Buenos Aires, Argentina; ^2^ Children and Adolescents Psychotherapy and Technology Lab (CAPT), Palo Alto University, Palo Alto, CA, United States; ^3^ Department of Psychology, International Institute for Internet Interventions i4Health, Palo Alto, CA, United States

**Keywords:** chatbot, artificial intelligence, conversational agent, parenting, user experience (UX), intervention

## Abstract

**Background:**

The use of chatbots to address mental health conditions have become increasingly popular in recent years. However, few studies aimed to teach parenting skills through chatbots, and there are no reports on parental user experience. Aim: This study aimed to assess the user experience of a parenting chatbot micro intervention to teach how to praise children in a Spanish-speaking country.

**Methods:**

A sample of 89 parents were assigned to the chatbot micro intervention as part of a randomized controlled trial study. Completion rates, engagement, satisfaction, net promoter score, and acceptability were analyzed.

**Results:**

66.3% of the participants completed the intervention. Participants exchanged an average of 49.8 messages (SD = 1.53), provided an average satisfaction score of 4.19 (SD = .79), and reported that they would recommend the chatbot to other parents (net promoter score = 4.63/5; SD = .66). Acceptability level was high (ease of use = 4.66 [SD = .73]; comfortability = 4.76 [SD = .46]; lack of technical problems = 4.69 [SD = .59]; interactivity = 4.51 [SD = .77]; usefulness for everyday life = 4.75 [SD = .54]).

**Conclusions:**

Overall, users completed the intervention at a high rate, engaged with the chatbot, were satisfied, would recommend it to others, and reported a high level of acceptability. Chatbots have the potential to teach parenting skills however research on the efficacy of parenting chatbot interventions is needed.

## Introduction

Behavior problems are prominent among children and adolescents (1, 2) and parenting programs have shown to be effective in reducing disruptive behaviors (3–6). However, many parents do not have access to such programs due to several barriers, such as a shortage of human therapists (7). Digital mental health interventions have become a popular method of overcoming the barriers to access and providing support for mental health. Parenting programs delivered through digital means have effectively treated behavioral problems in children and adolescents (8–12); however, the literature on chatbots for parenting skills is scarce.

Chatbots are computer-based programs that communicate with humans through text or voice conversations, are based on artificial intelligence (AI) and/or contain pre-programmed responses (13). The research on chatbots for mental health problems in adults has shown that chatbots can produce some unique effects compared to other digital interventions. Users are able to engage and bond with chatbots (14–16) and tend to humanize and perceive the chatbot as their friend (17). Users have found mental health chatbots to be helpful, informative, easy to use (13), and have reported that chatbots are more accepting and not as judgemental as humans (16). One study, in particular, found that users felt “loved” and “cared for” after using the chatbot (18). Additionally, chatbot studies reported significant improvements in depressive symptoms (19), anxiety symptoms (15), ADHD (20), and psychological distress (21). Throughout the onset of the COVID-19 pandemic, chatbots have been studied as a tool to offer psychological crisis support (22, 23).

The research on chatbots is still in its early stages, most chatbots are rule-based and follow scripts that are difficult to customize to each user (24). Some users have reported feeling frustration when the chatbot misunderstands them (25) and found it difficult to connect with them (26). Retaining and engaging users with chatbots is often challenging (14) and some people are still reluctant to use chatbots due to stigma (16, 26).

Analyzing user experience may help improve chatbots for mental health. Studies on user experience with chatbots have measured variables such as usability, satisfaction, engagement, and completion rate (27–29). Previous studies have looked at engagement by identifying the number of messages sent and characters typed by users during the conversation or over several days (30–32). Chatbots on user experience for parental interventions have undergone little research.

To our knowledge, there is one study on a parent training intervention delivered through a chatbot (33). The study examined the feasibility of delivering the beta version of a parenting chatbot micro intervention to teach parents how to praise their children. The intervention presented five skills for praising children effectively: defining the praise, being specific, avoiding combining praise with criticism, showing enthusiasm, and praising immediately. Seventy-eight percent of parents completed the intervention. On average, parents remembered 3.7 out of 5 taught skills and reported that they were likely to recommend the chatbot to other parents (7.44/10). During the micro intervention, parents sent an average of 54 messages, with a mean of 3 words per message. Overall, parents completed the intervention, were satisfied with it, and learned from the chatbot. While this suggests that parenting skills could be delivered *via* chatbots, some parents considered the chatbot's script impersonal or mechanical while others reported experiencing technical difficulties or they felt misunderstood by the chatbot.

This study aimed to assess the user experience of version 1.0 of the parenting chatbot micro intervention to teach how to praise children. Specifically, the study aimed to analyze the completion rate, messages sent, characters typed, degree of acceptability, satisfaction, and net promoter score.

## Methods

### Participants

Participants were recruited through Facebook posts and email list advertisements. A total of 170 people participated in the study. To be included in the study, participants had to reside in Argentina and have at least one child between the age of two to eleven years old.

### Materials and measures

**User Experience Questionnaire**. Similar to previous research (34, 35), five *ad hoc* questions were designed to address the acceptability: ease of use, comfort, lack of technical problems, interactivity, and usefulness for everyday life. Participants rated each question using a Likert scale from 1 (“strongly disagree”) to 5 (“strongly agree”).

**Satisfaction Questionnaire**. A Likert scale from 1 to 5 was used, with 1 being “Totally disagree” and 5 being “Totally agree”, to assess participants' satisfaction with the question “How satisfied are you with the intervention?”.

**Figure 1:**
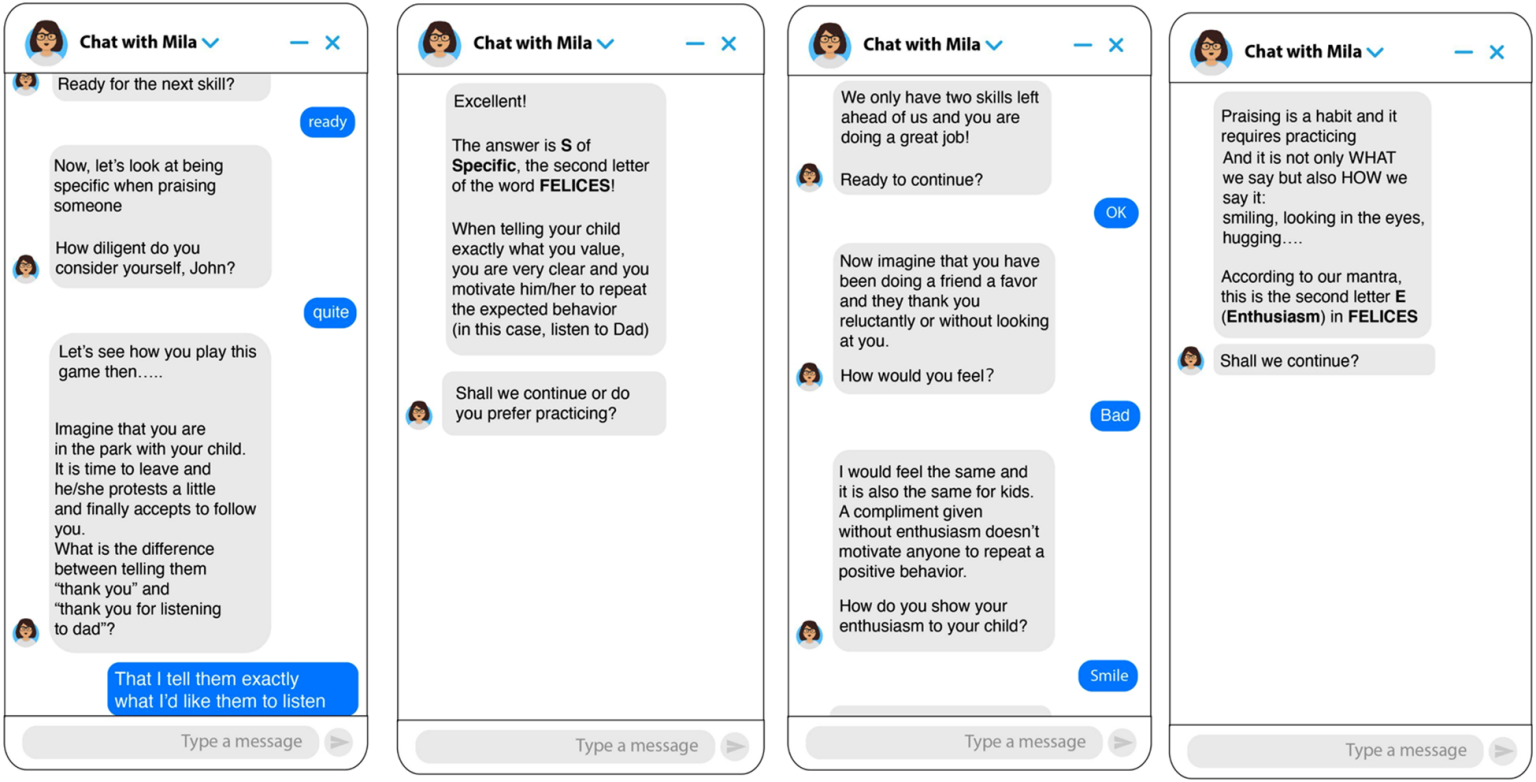
Screenshots of the chatbot for the “Specific” and “Enthusiasm” skills. Note. Images on the left show skill 1 (Specific) and images on the right show skill 4 (Enthusiasm).

**Figure 2:**
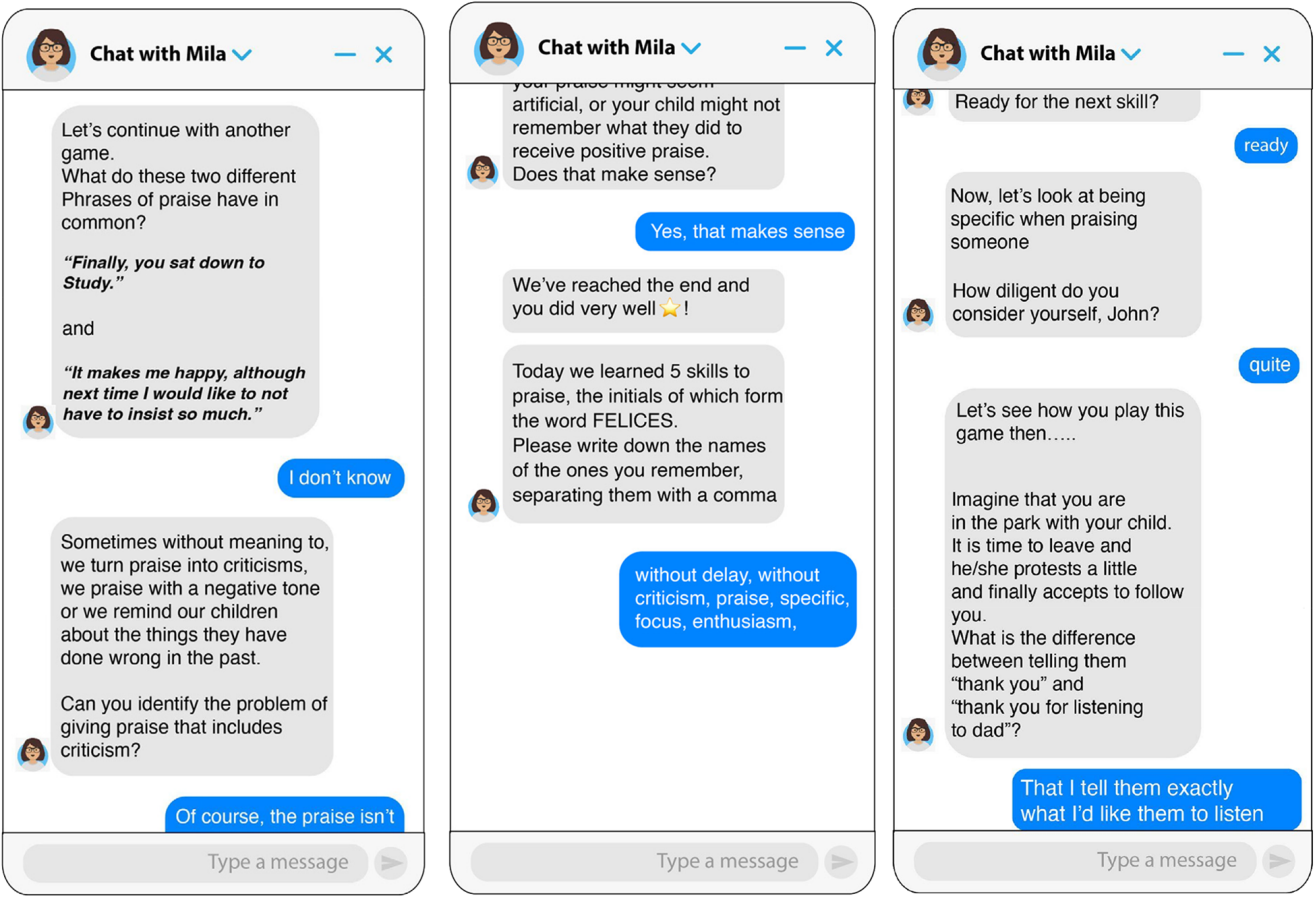
Screenshots of the chatbot for the “Avoid combining praise with criticism” skill and reviewing what was taught at the end of the intervention. Note. The image on the left shows skill 2 (Avoid combining praise with criticism) and the image on the right shows the review of what was taught at the end of the intervention.

**Chatbot Parenting Micro Intervention**. An intervention was designed based on an initial module of the Incredible Years parenting program (6). Its general objective is to teach parents to use positive attention and praise to stimulate positive behaviors in their children. An artificial intelligence software (TESS) designed by X2AI to provide mental health care was used. The intervention aimed to teach five skills for effective use of praise. The skills were grouped for presentation to participants under the acronym F.E.LIC.E.S. (“HAPPY” in Spanish - the original language of the intervention). The 5 skills taught were: Focus (choose specific behaviors you want to encourage), be specific, avoid combining praise with criticism, show enthusiasm when praising, and praise immediately after the good behavior. The intervention lasted approximately 15 min and was designed based on principles of conversational design, gamification, and study practices that promote interaction and learning. It included open-ended questions to encourage reflection, conversational games to distinguish between good and bad compliments, and a brief exam at the end to recover and consolidate the learning of the acquired information. Behavioral change techniques were employed, such as modeling, reinforcement, and practice. See Figures 1, 2 for samples of dialogue from the chatbot.

### Procedure

Participation was anonymous, voluntary, and unpaid. All participants were users of the social media on which the study was conducted (Facebook) prior to the start of the research. While using the social media during their daily life, they were presented with posts with a link to the chat feature in which to initiate the intervention, and an explanation on how to start the conversation with the chatbot. No training on how to use the chatbot was necessary as the chatbot was designed to explain the objectives, timelines and steps of the intervention during the first exchanges of the conversation. Furthermore, as participants were users of the platform, it was expected that they would have previous experience using the chat feature to engage in conversations with other people.

Once the conversation was initiated by the users, the chatbot explained the objectives and assessed the inclusion criteria. Those who met the inclusion criteria gave their consent electronically. All participants completed the baseline assessment and then were randomly assigned to an experimental group (parenting micro intervention) or control group one-day waitlist. After concluding the intervention, user experience and the level of satisfaction of the experimental group were evaluated. In line with ethical requirements in human research, all parents had access to the intervention after the study was completed. This study was approved by the Ethics Committee of the University of Buenos Aires, Argentina (CEI2120007).

### Data analysis

The descriptive sociodemographics variables were analyzed for the total sample of participants who started the study (initial sample). Analysis of the current study focus on the user experience of the participants in the experimental group. The completion rate was analyzed using the frequency and percentage of participants who completed each skill. Engagement with the intervention was analyzed using the average number of messages and characters sent. For the analyses related to the user experience, the descriptive characteristics of the variables (Satisfaction, Recommendation, Ease of use, Comfort, Absence of technical problems, Interactivity, and Usefulness in everyday life) were reported.

## Results

### Demographics

**Figure 3:**
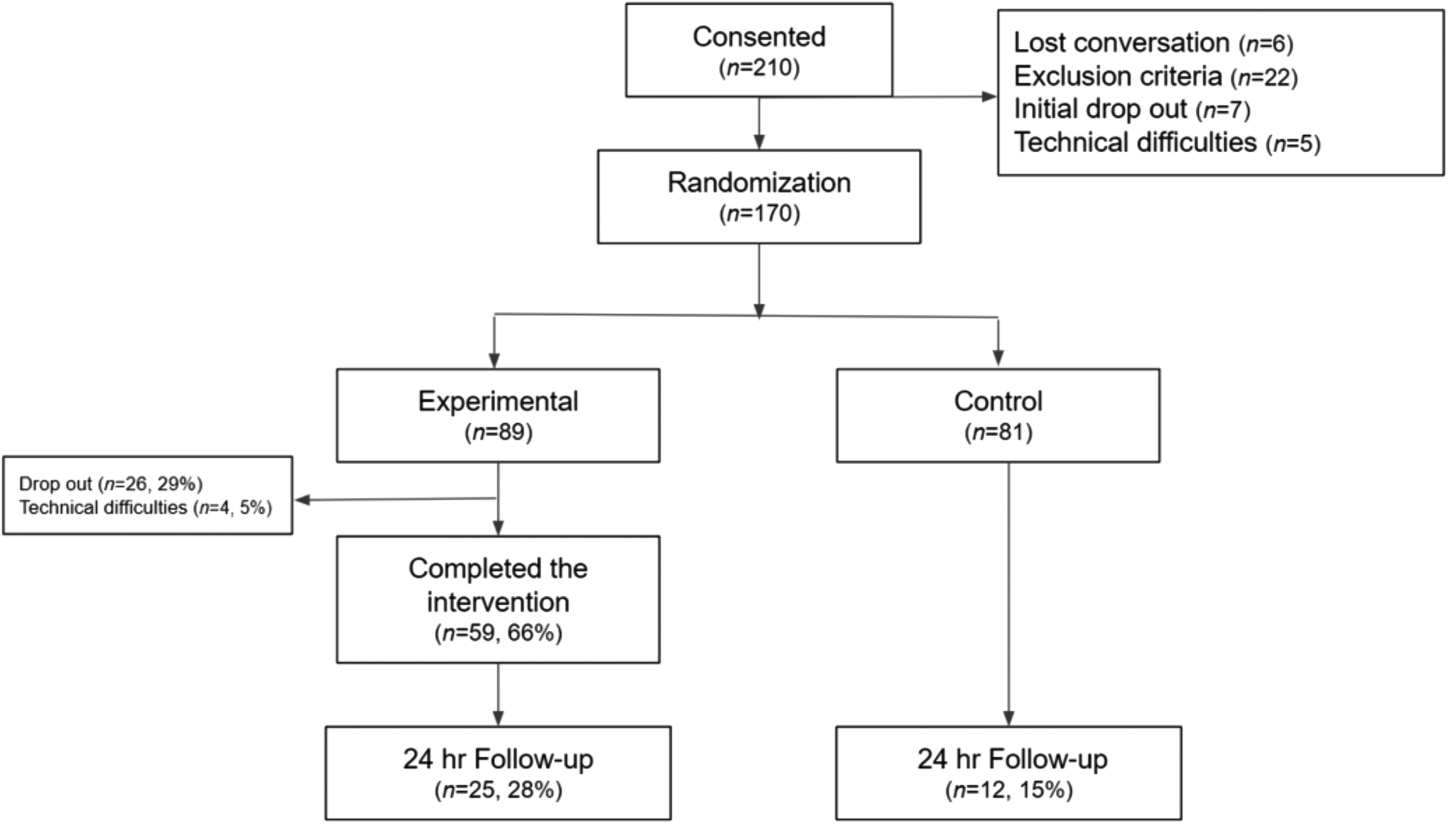
Flow of participants through the study.

The total sample consisted of 170 parents (*M_age_*_ _= 35.84; *SD* = 6.47). The majority of participants identified as female (*n* = 162, 95.3%), married (*n* = 119, 70%), had a university or tertiary level of education (*n* = 129, 75.9%), and were employed at the time of the study (*n* = 139, 81.7%). The average age of the parents' children was 5.69 years old (*SD* = 2.93), and there was relative gender homogeneity (51.2% girls and 48.8% boys). See Table 1. A total of 89 parents were randomly assigned to the experimental group and 81 to the control group. See Figure 3. There were no significant differences in demographic variables between the experimental and control group or between the intent-to-treat sample and completers. The findings presented in the following sections refer to the experimental condition only, and outcome analyses are reported elsewhere.

**Table 1:** Sociodemographic characteristics of baseline sample (*N* = 170).

		Frequency (percentage)
Gender	Female	162 (95.3%)
	Male	8 (4.7%)
Education Level	Primary	3 (1.8%)
	Secondary	27 (15.9%)
	University/technician	129 (75.9%)
	Other	11 (6.5%)
Marital Status	Single	9 (5.3%)
	Married	119 (70%)
	Divorced	11 (6.5%)
	Other	31 (18.2%)
Employment Status	Employed	82 (48.2%)
	Self-employed	57 (33.5%)
	Unemployed	31 (18.2%)
Child Gender	Female	87 (51.2%)
	Male	83 (48.8%)

### Completion rates

**Figure 4:**
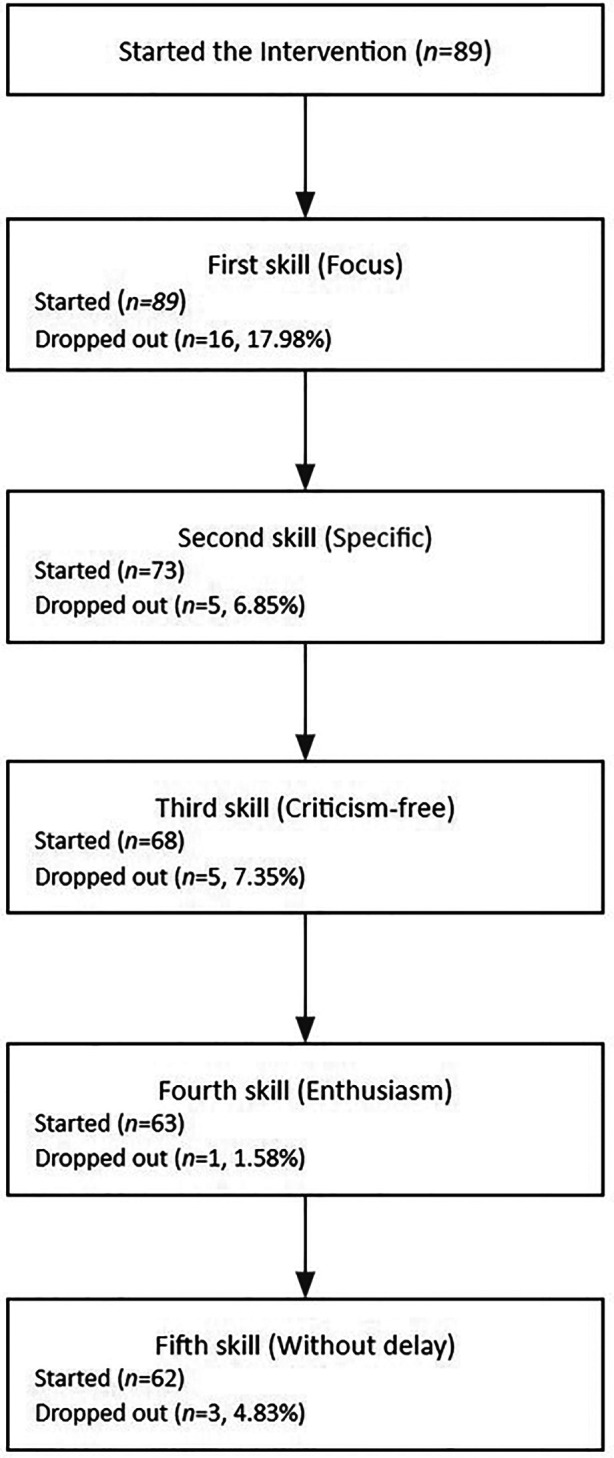
Skills completed.

The completion rate of the intervention in the experimental group was 66.3% (*N* = 59). The first skill had the lowest completion rate (82.02%), while all subsequent skills had a completion rate above 92%. See Figure 4 for a description of dropout by skill.

Throughout the study, the platform's policy regarding hosted chatbots was modified. A restriction on the frequency with which chatbots could reach out to users after a first conversation was introduced, leading to the automatic filtering of most messages sent 24 h after the end of the intervention.

### Engagement

Participants sent an average of 49.8 messages (range 20–80, *SD* = 1.53), an average of 660.9 characters (range 29–2398; *SD* = 51.68), and an average of 12.98 characters per message.

### Satisfaction and net promoter score

Participants provided an average satisfaction score of 4.19 (*SD* = .79) and reported that they would recommend the chatbot to other parents: Net promoter score was 4.63 (*SD* = .66) out of 5, with 5 being the highest.

### Acceptability

Parents provided a high level of acceptability (ease of use = 4.66 [*SD* = .73], comfort = 4.76 [*SD* = .46], lack of technical problems = 4.69 [*SD* = .59], interactivity = 4.51 [*SD* = .77], and usefulness for everyday life = 4.75 [*SD* = .54]). All dimensions measured obtained a mean greater than 4.51. The results indicated a high degree of usability and acceptability perceived by the participants who completed the intervention. See Table 2.

**Table 2:** Descriptive statistics of the variables related to user experience (*n* = 59).

Measurements	M (SD)	Median	Range	95% IC
Ease of use	4.66 (.73)	5	1–5	[4.47, 4.85]
Comfort	4.76 (.46)	5	3–5	[4.64, 4.88]
Absence of technical problems	4.69 (.59)	5	2–5	[4.31, 4.71]
Interactivity	4.51 (.77)	5	2–5	[4.33, 4.72]
Usefulness in everyday life	4.75 (.54)	5	3–5	[4.60, 4.89]

Note. A Likert scale from 1 to 5 was used, with 1 being “Totally disagree” and 5 “Totally agree”.

## Discussion

Chatbots represent a promising intervention for delivering digital mental health. However, research on the user experience of chatbots for parent training has been scarce. This study aimed to assess the user experience of Version 1.0 of a parenting chatbot micro intervention to teach how to praise children and compare these results with those of other mental health chatbots and the beta version of the current intervention (reported in a previous pilot study - (33).

A total of 66.3% of participants completed the intervention. This rate is nearly equal to that of another single-session, web-based, self-guided parenting intervention (36) [66.4%], and comparable to brief self-guided parenting studies utilizing other technologies, such as videos (37) [45%], podcasts (38) [71.9%]), and TV series (39) [65.4%]). Congruent with most literature on online interventions, the completion rate of the current study was lower than the one reported in human-supported digital parenting interventions (40). It was also lower compared to a preventively focused 2-hour face-to-face group discussion on how to manage disruptive behaviors (41). One-session face-to-face interventions have little chance for attrition, though their scalability is lower.

Interestingly, the completion rate doubled that of another single-session parenting intervention delivered contemporaneously in Finland, during the starting stage of the pandemic (42) [32.6%]. Authors have suggested that the high dropout rate may have been due to participants having several support channels, and because the pandemic was relatively controlled in Finland (42) This suggests that digital parental interventions may be most useful in countries such as Argentina, where parents have fewer support resources, and that it was offered at a time of great need.

Compared to the pilot study (33), the completion rate was lower (78% vs. 66.3%), though the present study included more participants and had a more diverse recruitment effort (online vs. snowball recruitment), making the results more generalizable. The current completion rate is higher than the one obtained in another chatbot study conducted in Argentina (40%), although that intervention took place over a longer period of time (8 weeks) (32). Overall, the completion rate of the current study was acceptable and promising, considering that participants did not receive incentives for completion or any human support, which is associated with increased adherence (27).

Regarding attrition rates the first skill (i.e.,: Focus, choose specific behaviors you want to encourage) accounted for more than half of the total drop-outs, and the following skills had considerable lower drop out rates. This first skill demanded more interactions than the subsequent skills, suggesting that more agile modules favor adherence. It has been suggested that participants in chatbot interventions tend to drop out in the first stages because they may not perceive a need for the intervention (43) or are requested to provide too much information (44). Since the majority of participants in the current study were recruited through a Facebook post with little information, some may have only fully understood the objectives of the intervention during the first skill and considered that they did not need it. Additionally, during the first skill, the chatbot asked about parental styles (e.g., “Which words do you usually use to praise your child?”), and some participants may not have felt comfortable sharing that kind of information and decided to drop out. Since the dropout rates were low in the subsequent skills, future iterations of this intervention should test a more agile version of the script aimed at teaching parents to choose specific behaviors they want to encourage.

In terms of engagement, participants sent an average of 49.8 messages during a one-time 15-minute intervention. The number of messages is similar to what was found in the study of the beta version of the parenting micro intervention (*M* = 54.24) (33). Interestingly, another study on a Spanish-speaking chatbot (32) also reported a high average number of messages sent (*M* = 116). Overall, previous Spanish-speaking chatbot studies conducted in Argentina (32, 33) along with the current one have shown a higher engagement than what was found in a longer English-speaking chabot study (*M *= 17.57 messages) (45). It is possible that participants in Argentina are more open to sharing their issues with a chatbot than English-speaking users. In the US, studies with Latinx individuals show greater openness to communicating through text and mobile messaging platforms (46). It is possible that Latinx users may be well suited for chatbot interventions. Finally, previous studies have reported that the higher number of messages sent by the user was associated with higher completion rates (47), and satisfaction with the chatbot (32). Thus, the high number of messages sent by participants in the current study is a positive sign of engagement.

Participants provided a high satisfaction score and reported that they were very likely to recommend the chatbot to other parents. These results were similar to those of other mental health chatbot studies (17, 33), and indicate a good experience among participants. Another chatbot specifically designed for parents of newborns about stress, sleep, and infant feeding had lower satisfaction scores (*M* = 3.81), perhaps due to many parents experiencing technical problems (46%; (48)) or the specific challenges of having a newborn. It is possible that the high satisfaction level of the current chatbot was associated with the high ease of use and low rate of technical problems. Chatbots that tend to repeat questions or do not understand the user's intention have been reported as a cause of user annoyance (49) and time constraints are frequently reported by parents as barriers to therapy (7). The short duration of the intervention may have promoted greater satisfaction by adjusting to their needs and not asking only a few questions. Therefore, brief chatbot conversations may be well suited for parents struggling with their children's behaviors.

Parents who completed the intervention reported a high level of acceptability for each of the items analyzed (i.e., ease of use, comfort, absence of technical problems, interactivity, usefulness in everyday life). These acceptability levels are comparable to those obtained from other digital parenting interventions such as podcasts (38), websites (50), training television (39), and chatbots for other mental health problems (51, 52). The conversational nature of the intervention may have contributed to this high level of acceptability. Previous studies have identified that parents (53) and young adults (54) value interactive digital interventions in which they can write and talk in the same way they do with humans. Furthermore, the study was conducted during the COVID-19 pandemic, which was associated with increased levels of parental distress and child misbehavior in 2021 (55). Therefore, it is possible that in such a context, an intervention of this kind addressed relevant issues through a flexible and convenient format. More specifically, users rated the intervention's usefulness for everyday life with an average of 4.75 of 5 (95%). Since disruptive behaviors are a prominent problem among children (1), it is possible that the content offered may have aligned well with the interests of the participants in general and in the specific context of the COVID-19 pandemic.

### Limitations and future directions

The chatbot designed for the present study used a rule-based model method of input processing and response generation, in which the responses available in the system are chosen based on a fixed set of predefined rules. This model is more vulnerable to user spelling and grammatical errors, and less flexible than retrieval-based and generative models, which use machine learning algorithms and deep learning techniques (56). Sörensen (57) found that error handling and the ability to understand text are crucial in chatbot user perception, which is often difficult to achieve in rule-based models. Therefore, a future iteration of this intervention using more advanced processing systems could improve the participants' experience.

The current study used a one-time micro intervention and measured engagement based on the number of interactions the participants had with the chatbot. While this is a widely used metric in chatbot research, the findings are not generalizable to a whole chatbot-based treatment with multiple sessions. Thus, the engagement with the chatbot across time is unknown. Future studies should assess users' engagement with consecutive chatbot micro-interventions for parenting and incorporate the number of sessions completed as an additional engagement metric.

Completion rates, engagement, and acceptability scores suggest that the participants' experience was positive. However, this does not imply that the intervention has proven to produce significant changes, nor evidence of clinical efficacy and safety. Therefore, results should be read with caution and further research is needed before offering the intervention to a wider public.

Finally, more than ninety percent of the sample identified as female, thus these findings may not be generalizable to fathers. Since, the involvement of both parents tends to increase the outcomes of mental health treatments in children (58), interventions that can facilitate access to both parents are needed. The chatbot nature of this intervention could encourage the involvement of both parents regardless of their gender, and future studies should aim to recruit a more representative sample of parents. Furthermore, most participants had a high level of education so the results are not generalizable to participants with a lower level of education. This limits the generalizability of the results to this population. It is possible that the recruitment materials (e.g., text and photos of Facebook posts) were not sufficiently attractive or clear to populations with lower levels of education. Future studies should be designed to capture the attention and interest of more diverse populations, either by changing the recruitment materials and/or the recruitment channels.

## Conclusions

Behavior problems in childhood are highly prevalent, and there is a need to develop novel forms of parent training. Chatbot interventions could increase the accessibility of parent skills training. The results of the present study suggest that parents find a parenting chatbot micro intervention acceptable, have a positive user experience, were engaged, and were highly satisfied with the chatbot. Parents reported that they would recommend the chatbot to other parents and found it useful for everyday life. Chatbot intervention may align well with the participants' interests (especially during a time of need, such as the COVID-19 pandemic). Future studies should focus on the efficacy of the intervention by measuring if parents learn the skills, improve their parenting self-efficacy and decrease disruptive behavior in their children.

## Data Availability

The raw data supporting the conclusions of this article will be made available by the authors, without undue reservation.
